# The organic cation transporters 1 and 2 mediate ethanolamine cellular efflux and control systemic phosphatidylethanolamine level

**DOI:** 10.1016/j.jbc.2025.110911

**Published:** 2025-11-05

**Authors:** Julia Schubert, Ferhat Koca, Francesca Barone, Giuseppe Corona, Giuliano Ciarimboli, Michele Visentin

**Affiliations:** 1 Department of Clinical Pharmacology and Toxicology, University Hospital Zurich, University of Zurich, Zurich, Switzerland; 2 Department DiBEST (Biologia, Ecologia, Scienze della Terra) Unit of Biochemistry and Molecular Biotechnology, University of Calabria, Rende, Italy; 3 Immunopathology and Cancer Biomarkers Unit, IRCCS Centro di Riferimento Oncologico di Aviano (CRO), Aviano, Italy; 4 Experimental Nephrology, Medicine Clinic D, University Hospital Muenster, Muenster, Germany

**Keywords:** drug transport, lipid metabolism, lipid synthesis, membrane transport, metabolomics

## Abstract

The organic cation transporters (OCTs) OCT1 on the basolateral membrane of enterocytes and hepatocytes and OCT2 on the basolateral membrane of proximal tubular cells are essential in regulating systemic micronutrient levels, while also safeguarding tissues by preventing the buildup of potentially harmful endogenous metabolites, drugs, and xenobiotics. In the present work, we integrated *in vivo* comparative metabolomics and lipidomics analyses of serum from WT and Oct1/2^−/−^ mice with *in vitro* uptake measurements in HEK293 cells overexpressing OCT1 or OCT2, to identify and characterize novel endogenous substrates of OCT1/2. Among the significant metabolite changes, ethanolamine in the serum of Oct1/2^−/−^ mice was approximately 70% lower than in WT mice. The ethanolamine influx K_t_ mediated by OCT1/2 ranged from 7.6 ± 3.7 mmol/L (mouse Oct2) to 13.4 ± 8.1 mmol/L (mouse Oct1). OCT1/2 did not transport ethanolamine at physiologically relevant extracellular concentrations (10–100 μmol/L), suggesting that OCTs do not play a role in the hepatic/renal uptake of ethanolamine. Conversely, the release of ethanolamine by cells pre-exposed to ethanolamine at the extracellular concentration of 50 μmol/l was significantly greater in the presence of OCTs. Finally, the serum of the Oct1/2^−/−^ mice was characterized by a stark elevation across phosphatidylethanolamine and lysophosphatidylethanolamine species, but not in phosphatidylcholine and diacylglycerol species. Taken together, our *in vitro* and *in vivo* data indicate that mouse Oct1 and Oct2 are essential for facilitating the exit step of free ethanolamine vectorial transport and indirectly control systemic phosphatidylethanolamine level.

Humans possess seven characterized organic cation transporters (OCTs) within the solute carrier (SLC) 22 family, including OCT1-3, OCTN1-2, OCT6 (1), and more recently, CT2 (SLC22A16) and SLC22A15 transporter (2, 3), all featuring 12 transmembrane domains with one large extracellular loop and intracellular N and C termini (4, 5). OCT substrates are primarily amines—primary, secondary, tertiary, or quaternary—that are positively charged or zwitterionic at physiological pH, and their transport across cell membranes is driven by membrane potential and electrochemical gradients. The process favors cation uptake due to the inside-negative membrane potential, facilitating inward transport, but efflux can occur if intracellular substrate concentrations build up sufficiently to overcome this electrochemical driving force, allowing cations to exit the cell (6).

OCT1 and OCT2 show the broadest range of substrates, including several widely prescribed drugs, such as anticancer drugs—platinum-derivatives, tyrosine kinase inhibitors—the antidiabetic metformin, and the antibiotic gentamicin (7, 8). Because of the high expression level at key cellular membranes—OCT1 at the basolateral membrane of enterocytes and hepatocytes (9, 10), and OCT2 at the basolateral membrane of proximal tubular cells (11, 12)—they are considered major molecular determinants in the absorption, distribution, excretion, and potential adverse reactions of cationic drugs, which accounts for approximately 40% of approved medications. Individuals with low-function OCT1 variants exhibit decreased clearance of substrates such as morphine, fenoterol, and sumatriptan (13, 14, 15, 16), and a diminished response to metformin (17), indicating altered drug distribution and efficacy. Likewise, carriers of the OCT2 Ala270Ser transport-defective variant have a reduced risk of cisplatin-induced ototoxicity and nephrotoxicity due to decreased OCT2 activity (18). Regulatory guidelines currently mandate *in vitro* testing of new drugs for OCT2 inhibition (mandatory) and OCT1 inhibition (recommended) to anticipate potential drug interactions affecting renal and hepatic clearance pathways, ensuring a safer clinical drug development (http://www.ema.europa.eu/docs/en_GB/document_library/Scientific_guideline/2012/07/WC500129606.pdf) (http://www.fda.gov/Drugs/GuidanceComplianceRegulatoryInformation/Guidances/default.htm).

The tissue-specific expression of OCT1 and OCT2 (9, 10, 11, 12) underscores their crucial role in regulating systemic micronutrient levels, such as choline, essential for metabolic processes, while also preventing the buildup of potentially harmful metabolites like trimethylamine N-oxide, thereby maintaining metabolic homeostasis and protecting against related disorders (*e.g.*, cardiovascular disease) (19, 20, 21). The association of reduced-function polymorphisms in *SLC22A1*, encoding OCT1, with elevated plasma levels of LDL, total cholesterol, and triglycerides, highlights OCT1's potential role in lipid metabolism regulation (22). Accordingly, loss of Oct1 increases the AMP/ATP ratio, activating the AMP-activated protein kinase and lowering hepatic triglyceride levels in both healthy and leptin-deficient mice, suggesting that OCT1 plays a critical role in thiamine-dependent lipid and glucose metabolism, with implications for metabolic syndrome pathophysiology. Additionally, it is proposed that one mechanism by which metformin exerts its antidiabetic effects may involve inhibiting thiamine OCT1-mediated uptake in the liver, thereby influencing metabolic pathways regulated by thiamine and AMP-activated protein kinase activation (22, 23). Understanding the physio-pathological roles of OCT1 and OCT2 is crucial for elucidating their influence on drug pharmacokinetics and pharmacodynamics, to finally enhance our confidence to identify potential toxicity risks, thereby advancing drug safety profiles. The study aimed to discover new endogenous substrates of human (hereinafter, OCT1/2) and mouse (hereinafter, Oct1/2) OCT1 and OCT2, by integrating *in vivo* comparative metabolomics and lipidomics analyses with *in vitro* transport assays, thereby enhancing understanding of their substrate specificity and physiological roles.

## Results

### Ethanolamine level is reduced in the serum of Oct1/2^−/−^ mouse

**Figure 1:**
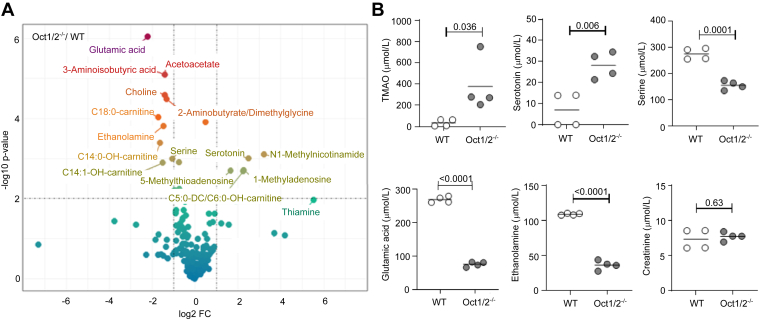
**Metabolomic changes in the serum of Oct1/2^−/−^ mouse.***A*, volcano plot comparing the 196 metabolites identified by LC–MS/MS in the serum of Oct1/2^−/−^ and WT mice. The fold change of concentration (log2 FC) is reported on the *x*-axis, the significance (−log10 *p* value) on the *y-*axis. The *vertical and horizontal dotted lines* show the cutoff of fold change = ±1, and of *p* value = 0.01, respectively. *B*, scatter dot plots of the absolute abundance of select metabolites. In all scatter dot plots, absolute levels of each metabolite relative to an internal standard are shown. Each dot represents one individual sample. Comparisons of the means were performed by unpaired Student's *t* test. OCT, organic cation transporter.

To identify novel endogenous substrates of OCT1/2, we performed a comprehensive metabolomics analysis of the serum from WT and Oct1/2^−/−^ mice. Twelve-week-old WT and 23-week-old Oct1/2^−/−^ mice were used. We included age-matched controls—10-week-old WT and 25-week-old Oct2^−/−^ male mice—to account for age-related variations. The expression of Oct1 in OCT2^−/−^ mice maintains normal tubular secretion and hepatic uptake of most substrates, owing to the overlapping substrate specificity between Oct1 and Oct2, which compensates for the loss of Oct2 function in these tissues (24). Among the known substrates of Oct1 and Oct2, serotonin (Fig. 1, *A* and *B*), N1-methylnicotinamide (Fig. 1*A*), and trimethylamine N-oxide (Fig. 1*B*) were found elevated in Oct1/2^−/−^ mice, whereas choline level was significantly higher in the WT mice (Fig. 1*A*). Conversely, the level of creatinine, which has been previously shown to be partially secreted *via* Oct2 (25), was comparable between the groups (Fig. 1*B*). We found that ethanolamine, which is structurally similar to choline, and exists primarily as a monocation at pH 7.4, was significantly reduced in the serum of Oct1/2^−/−^ mice. Absolute quantification analysis confirmed that the serum level of ethanolamine in Oct1/2^−/−^ mice was approximately 70% lower than in WT mice (36.1 ± 6.64 *versus* 108.6 ± 1.32 μmol/L, *p* < 0.0001) (Fig. 1*B*). The smaller ethanolamine reduction (∼20%) seen in the serum of Oct2^−/−^ mice compared to the respective WT mice (Fig. S1) confirmed that these effects are specifically due to Oct1/2 deficiency and not confounded by age disparities.

### Mouse and human OCT1 and OCT2 are low-affinity carriers of ethanolamine

**Figure 2:**
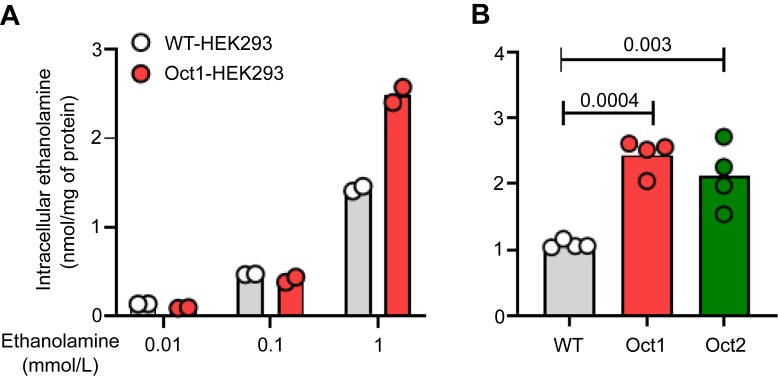
**Ethanolamine uptake in HEK293 cells overexpressing mouse Oct1 or Oct2.***A*, one-minute uptake of ethanolamine at the indicated extracellular concentration, in WT-HEK293 and mOCT1-HEK293 cells. Results are expressed as the mean from one representative experiment performed in duplicate. *B*, one-minute uptake of ethanolamine at the extracellular concentration of 1 mmol/L, in WT-, Oct1-, and Oct2-HEK293 cells. Uptake data are expressed as the mean from four independent experiments. The indicated *p* values were calculated from one-way ANOVA test followed by Dunnett’s multiple comparisons test. HEK, human embryonic kidney; OCT, organic cation transporter.

**Figure 3:**
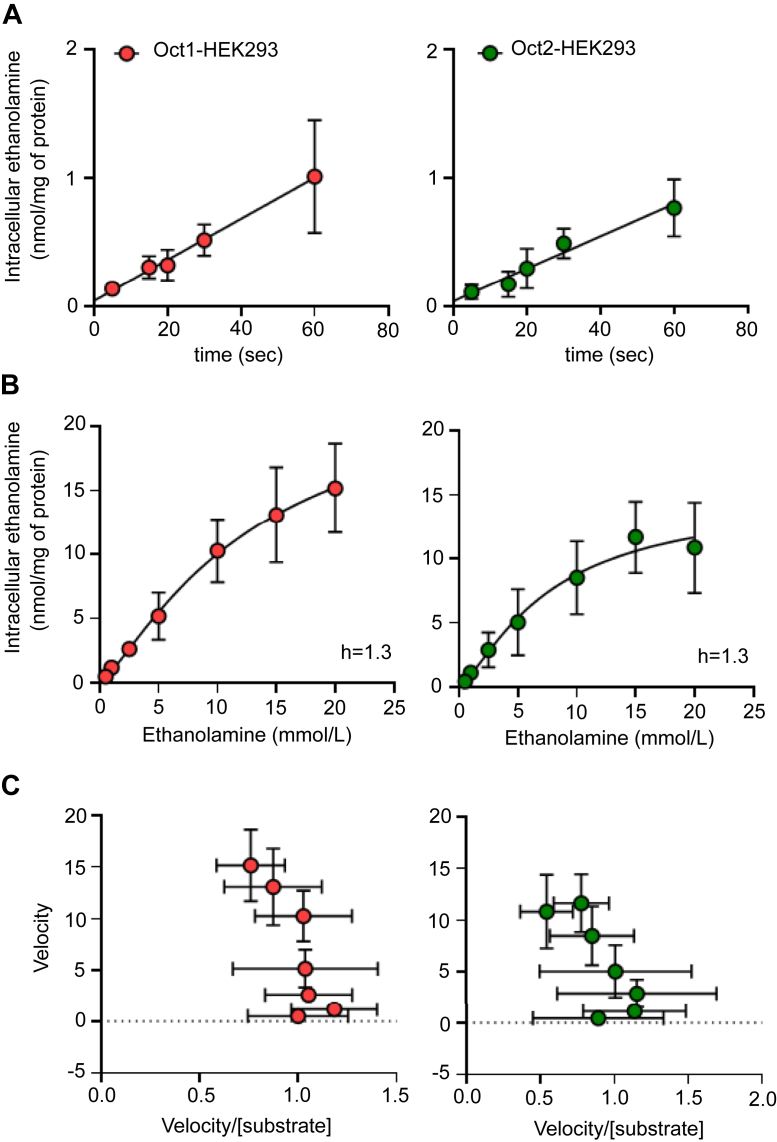
**Kinetic analysis of ethanolamine influx in mouse Oct1-and Oct2-HEK293 cells.***A*, time course of uptake of 1 mmol/L ethanolamine mediated by Oct1 and Oct2. Data were corrected for uptake in WT-HEK293 cells. Data are the mean ± SD from three independent experiments, each performed in duplicate. *B*, one-minute uptake of increasing extracellular concentrations of ethanolamine by Oct1 and Oct2. All data were subtracted of the uptake values measured in the WT-HEK293 cells. Line was best fit to an allosteric sigmoidal equation Y = V_max_∗X^h^/(K_prime_ + X^h^), where K_prime_ is an estimation of K_m_, V_max_ is the maximum capacity, and h is the Hill slope. *C*, Eadie–Hofstee transformation of the kinetic data. Kinetics experiments were performed three times, each in duplicate. HEK, human embryonic kidney; OCT, organic cation transporter.

**Figure 4:**
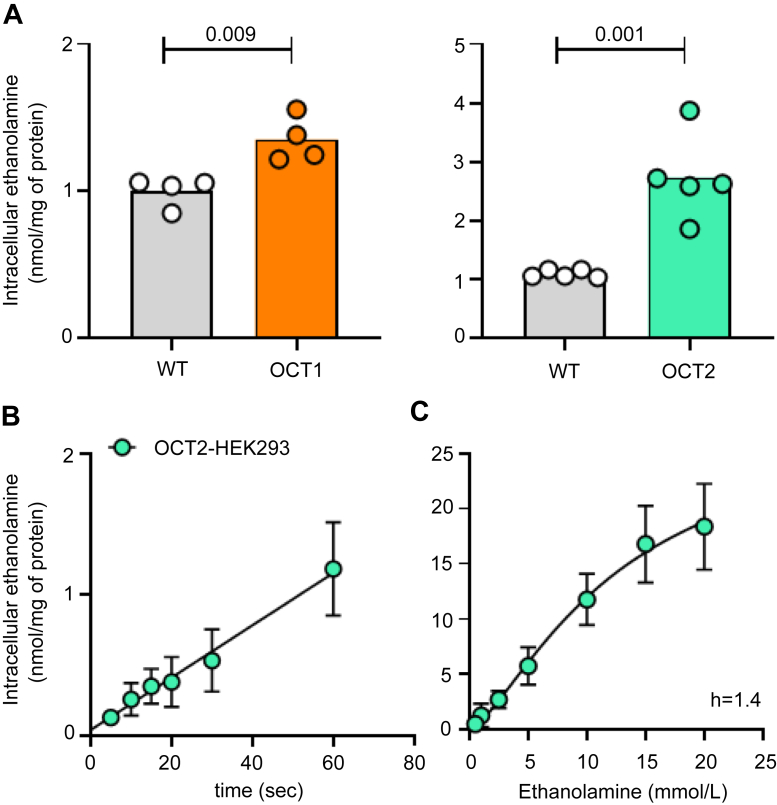
**Ethanolamine uptake by human OCT1 and OCT2.***A*, one-minute uptake of ethanolamine at the extracellular concentration of 1 mmol/L, in WT-, OCT1-, and OCT2-HEK293 cells. In scatter plots, each data point represents one independent experiment. The indicated *p* values were calculated from unpaired Student's *t* test. *B*, time course of uptake of 1 mmol/L ethanolamine mediated by OCT2. Data were corrected for uptake in WT-HEK293 cells. Data are the mean ± SD from three independent experiments, each performed in duplicate. *C*, one-minute uptake of ethanolamine by OCT2 as a function of the extracellular concentration of substrate. All data were subtracted of the uptake values measured in the WT-HEK293 cells and expressed as the mean ± SD from three independent experiments, each performed in duplicate. Line was best fit to an allosteric sigmoidal equation Y = V_max_∗X^h^/(K_prime_ + X^h^), where K_prime_ is an estimation of K_m_, V_max_ is the maximum capacity, and h is the Hill slope. HEK, human embryonic kidney; OCT, organic cation transporter.

Preliminary experiments with Oct1 showed that at extracellular concentrations matching those measured in the mouse serum (0.01–0.1 mmol/L, Fig. 1*B*) the uptake of ethanolamine in WT-human embryonic kidney (HEK) 293 and Oct1-HEK293 cells was comparable (Fig. 2*A*). Only when the uptake was assessed at the extracellular concentration of 1 mmol/L, the intracellular level of ethanolamine was significantly higher in Oct1-HEK293 (2.44 ± 0.26 nmol/mg of protein) and Oct2-HEK293 cells (2.12 ± 0.49 nmol/mg of protein) than in the WT-HEK293 cells (1.08 ± 0.06 nmol/mg of protein) (Fig. 2*B*). To establish an interval over which ethanolamine uptake was unidirectional under these conditions, the time course of ethanolamine uptake was assessed over 1 min at an extracellular concentration of 1 mmol/L. It can be observed that uptake of ethanolamine mediated by Oct1 or Oct2 as a function of time over this interval was constant with a slope that intercepts approximately the point of origin at zero time, reflecting the influx phase of the uptake process (Fig. 3*A*). The influx of ethanolamine as a function of the extracellular concentration of substrate by Oct1 or Oct2 was assessed. Figure 3*B* illustrates the nonlinear regression curve of ethanolamine influx. The line was best fit to the sigmoidal equation with a calculated hill slope of 1.3, indicating a weak positive allosterism. The concave curvature towards the *y*-axis of the Eadie–Hofstee plot of the data (Fig. 3*C*) is consistent with a positive allosterism. The K_t_ for Oct1 and Oct2 was computed to be 13.4 ± 8.1 mmol/L and 7.6 ± 3.7 mmol/L, respectively (Table 1). The analysis was extended to human OCT1 and OCT2. Figure 4*A* shows that at the extracellular concentration of 1 mmol/L, ethanolamine intracellular accumulation in HEK293 cells overexpressing OCT1 was 35% higher than that in WT-HEK293 cells (1.35 ± 0.15 *versus* 1.00 ± 0.10 nmol/mg of protein, *p* = 0.009). The intracellular level of ethanolamine in OCT2-HEK293 cells was approximately three times higher than that in the WT-HEK293 cells (2.74 ± 0.72 *versus* 1.10 ± 0.06 nmol/mg of protein, *p* = 0.001). The influx kinetics of ethanolamine for OCT2 was assessed over 1 min, an interval reflecting the unidirectional transport of ethanolamine into the cells (Fig. 4*B*). Similarly to mouse Oct1 and Oct2, human OCT2-mediated uptake of ethanolamine was characterized by a weak positive cooperativity (h = 1.4) (Fig. 4*C*), with a K_t_ of 12.7 ± 5.3 mmol/L (Table 1). Albeit significant, the measured uptake value of ethanolamine in human OCT1-HEK293 cells was considered insufficiently high to reliably determine the influx kinetics of ethanolamine *via* human OCT1.

**Table 1:** Ethanolamine influx kinetics

Kinetic parameters	Oct1	Oct2	OCT2
	Mean ± SE	Mean ± SE	Mean ± SE
K_t_ (mmol/L)	13.4 ± 8.1	7.6 ± 3.7	12.7 ± 5.3
V_max_ (nmol/mg of protein/min)	24.4 ± 8.8	14.9 ± 3.9	28.6 ± 7.7
Hill slope	1.3 ± 0.3	1.3 ± 0.4	1.4 ± 0.3

K_t_ and V_max_ were calculated from the nonlinear regression equation Y = V_max_∗X^h^/(K_t_^h^ + X^h^).

Results are expressed as the mean and standard error of the mean from three independent experiments, each performed in duplicate.

Oct1, mouse organic cation transporter 1; Oct2, mouse organic cation transporter 2; OCT2, human organic cation transporter 2.

### Mouse and human OCT1 and OCT2 regulate ethanolamine efflux

**Figure 5:**
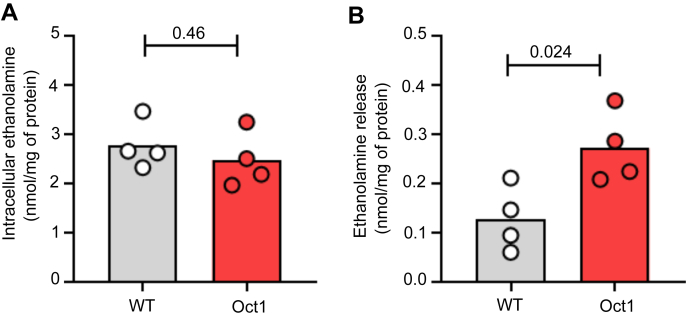
**Analysis of ethanolamine efflux mediated by mouse Oct1.***A*, intracellular ethanolamine was measured in Oct1- and WT-HEK293 cells incubated for 30 min with ethanolamine at the extracellular concentration of 50 μM (C_i_). *B*, intracellular ethanolamine in Oct1- and WT-HEK293 in which the 30 min with ethanolamine at the extracellular concentration of 50 μM was followed by a 10-s washout in ethanolamine-free buffer (C_F_). The ΔC (C_i_-C_F_) represents the amount of ethanolamine released over 10 s. In scatter plots, each data point represents one independent experiment. Comparisons of the means were performed by unpaired Student's *t* test. HEK, human embryonic kidney; OCT, organic cation transporter.

Experiments in Oct1-HEK293 cells indicate that OCTs do not transport ethanolamine at extracellular concentrations matching the plasma level (0.01–0.1 mmol/L) (Fig. 2*A*), and that OCTs do not play a role in the uptake of ethanolamine. Meanwhile, the Oct1/2^−/−^ mouse displayed a significantly lower circulating level of ethanolamine than the WT, consistent with a role of OCTs in the release of ethanolamine from the liver and kidneys into the circulation. According to this paradigm, OCT overexpressing cells exposed to physiological extracellular concentrations of ethanolamine are expected to have a comparable intracellular accumulation and a faster efflux rate than WT cells. This turned out to be the case. WT-HEK293 and Oct1-HEK293 cells incubated for 30 min with ethanolamine at the extracellular concentration of 50 μmol/L showed a comparable intracellular level of substrate (2.76 ± 0.49 *versus* 2.47 ± 0.56 nmol/mg of protein, *p* = 0.46) (Fig. 5*A*). When the 30-min incubation was followed by a 10-s washout, the intracellular level of ethanolamine in the WT-HEK293 cells was higher than that in the Oct1-HEK293 cells. The calculated release of ethanolamine by Oct1-HEK293 cells was significantly greater than that in the WT-HEK293 cells (0.27 ± 0.07 *versus* 0.13 ± 0.07 nmol/mg of protein, *p* = 0.026) (Fig. 5*B*).

### Phosphatidylethanolamine and lysoPE level is elevated in the serum of Oct1/2^−/−^ mouse

**Figure 6:**
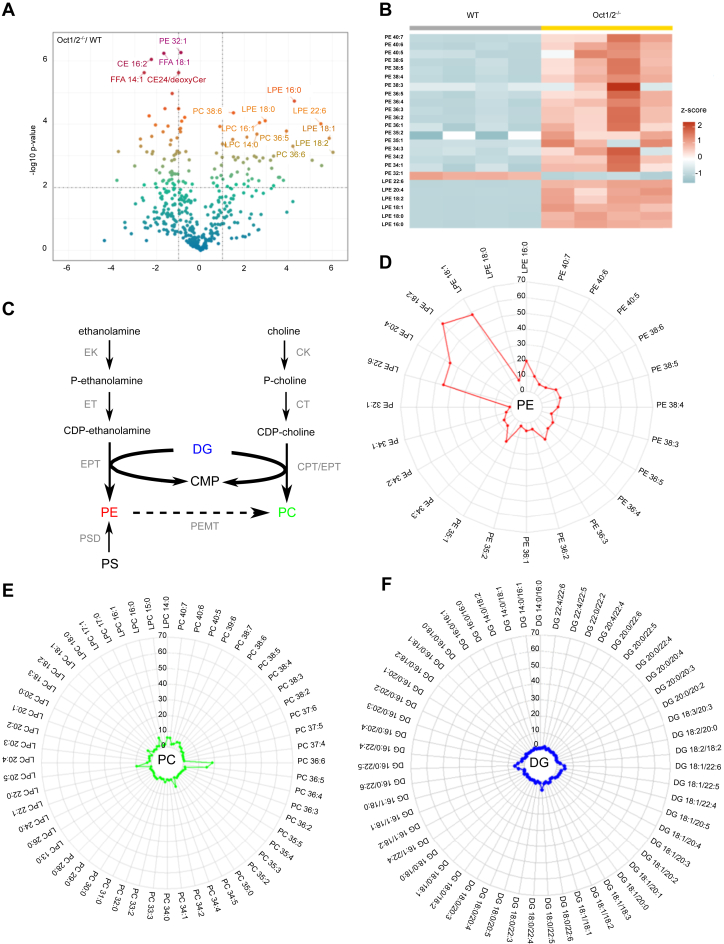
**Oct1/2-associated circulating lipid signature shift.***A*, volcano plot comparing the 221 lipid species identified by LC–MS/MS in the serum of Oct1/2^−/−^ and WT mice. The fold change of concentration (log2 FC) is reported on the *x*-axis, the significance (−log10 *p* value) on the *y*-axis. The *vertical and horizontal dotted lines* show the cutoff of fold change = ±1, and of *p* value = 0.01, respectively. *B*, heat map cluster analysis of the corrected phosphatidylethanolamine (PE) and lysophosphatidylethanolamine (LPE) species in the serum of Oct1/2^−/−^ and WT mice. *C*, the CDP–ethanolamine pathway parallels the CDP–choline pathway. Ethanolamine and choline are phosphorylated to phosphoethanolamine (P-ethanolamine) and phosphocholine (P-choline) by the ethanolamine (EK) and choline kinases (CKs), respectively. CTP:phosphoethanolamine cytidylyltransferase (ET) and CTP:phosphocholine cytidylyltransferase (CT) converts P-ethanolamine and P-choline to CDP-ethanolamine/choline. The final step in the pathways is catalyzed by the CDP-choline:1,2-diacylglycerol choline phosphotransferase (CPT) and the CDP-ethanolamine:1,2-diacylglycerol choline/ethanolamine phosphotransferase (EPT) to generate PE and PC. The alternative pathway for PE synthesis is mediated by the phosphatidylserine (PS) decarboxylase (PSD). In the the liver, PE can be converted to PC by the phosphatidylethanolamine N-methyltransferase (PEMT). *D*-*F*, radar plot of ethanolamine- and choline-derived lipid species and DG species. Each axis represents a lipid species, and the length of each axis corresponds to the fold change between the values measured in the Oct1/2^−/−^ relative to those measured in the WT mice. OCT, organic cation transporter.

The reduction in serum ethanolamine observed in Oct1/2^−/−^ mice was accompanied by a pronounced increase in phosphatidylethanolamine (PE) and lysoPE (LPE) species across various chain lengths, saturation levels, and modifications (Fig. 6, *A* and *B*). The comparable PE profile between WT and Oct2^−/−^ C57BL/6 male mice (Fig. S2), rules out an age-related effect. The CDP–ethanolamine pathway carries out the *de novo* synthesis of PE, which parallels CDP–choline pathway for phosphatidylcholine (PC) *de novo* synthesis (26) (Fig. 6*C*). For most of the PE and LPE species, the level in the serum of the Oct1/2^−/−^ mice was at least 5 times higher than that measured in the WT mice (Fig. 6*D*). Despite a marked reduction in serum choline levels in Oct1/2^−/−^ mice compared to WT mice (Fig. 1*A*), the majority of PC and lysophosphatidylcholine species remained stable (Fig. 6*E* and Table S1), suggesting that CDP–ethanolamine pathway is more dependent than CDP–choline pathway on substrate availability. Meanwhile, the serum level of diacylglycerol (DG) species, a crucial lipid sitting at the intersection of various lipid metabolism pathways, including PE *de novo* synthesis, remained unchanged (Fig. 6*F* and Table S1). Taken together, our data suggest that the PE elevation in the serum of the Oct1/2^−/−^ mice is arguably driven by an increased ethanolamine intracellular availability.

## Discussion

Studies in Y79 human retinoblastoma cell cultures (27), rabbit retina (28), hamster heart (29), bovine aortic endothelial cells (30), and human placental brush-border membrane vesicles (31) indicated that ethanolamine uptake occurs by both a high-affinity and sodium-dependent as well as a low-affinity and sodium-independent mechanism, but the membrane proteins involved are yet to be defined. In the present work, we demonstrate that OCT1 and OCT2 are low-affinity transporters of ethanolamine, thereby providing a clearer understanding of the molecular components mediating ethanolamine transport across cell membranes. When screening substrates for bidirectional transporters like OCT1/2, it is essential to consider both extracellular and intracellular concentrations at equilibrium to establish physiologically relevant screening conditions, rather than solely relying on extracellular levels measured in body fluids. If OCT1/2 had only been screened at the ethanolamine concentration measured in the mouse serum (10–100 μmol/L), no activity would have been detected (Fig. 1*B*). However, considering the pK_a_ of the amine group being around 8.5—at pH 7.4 the ethanolamine molecule is mostly protonated (z = +1)—and the negative resting membrane potential, the intracellular concentration of ethanolamine can be estimated to be roughly 10 times higher than the extracellular one. The estimate aligns with prior research indicating serum and intrahepatic ethanolamine levels in rats of approximately 30 μmol/L and 30 μmol/g, respectively, translating to an intracellular concentration of around 540 μmol/L (32)—a concentration at which ethanolamine was transported by OCT1/2 in our experiments (Figs. 2*B* and 4*A*)—and with the significantly different rate of efflux measured in Oct1-HEK293 and WT-HEK293 cells despite the comparable intracellular concentration of ethanolamine (Fig. 5). Taken together, our *in vitro* and *in vivo* data indicate that OCTs, predominantly located on the basolateral membrane of the polarized epithelia of the intestine, liver, and kidneys (8), are essential for facilitating the exit step of the vectorial transport of dietary ethanolamine, as well as that obtained from the hepatic PE degradation.

In the cell, ethanolamine is phosphorylated by the ethanolamine kinase to phosphoethanolamine, which is the substrate of the CTP:phosphoethanolamine cytidylyltransferase (ET), to obtain the high-energy intermediate CDP-ethanolamine (Fig. 6*C*). CDP-ethanolamine:1,2-diacylglycerol ethanolamine phosphotransferase catalyzes the final reaction of the pathway, using CDP-ethanolamine and a lipid, typically DG to form PE. Phosphoethanolamine cytidylyltransferase is considered the pace-setting enzyme of PE synthesis, as overexpression of the ethanolamine kinase in mammalian cells does not alter the steady-state levels of PE but only leads to an accumulation of glycerol-phosphoethanolamine (33). However, studies in rat livers and primary cultured hepatocytes indicate that at extracellular concentrations ranging from 10 to 50 μmol/L, ethanolamine supply dictates PE *de novo* synthesis, and that the total availability of DG and its fatty acid composition may significantly affect the rate of PE (32, 34, 35). It is plausible, albeit not shown, that the reduced circulating level of free ethanolamine in Oct1/2^−/−^ mice results in the raise of the intracellular ethanolamine concentration, which, in turn, drives PE synthesis and release. Moreover, the comparable level in serum DG species suggests that intracellular ethanolamine availability rather than lipid metabolism alterations underlines the stark increase in serum PE species. PE synthesis follows the enzymatic reactions that constitute the biosynthetic pathway of PC (26); however, the reduced choline concentration measured in the serum of Oct1/2^−/−^ mice was not associated with increased circulating PC species. This is in line with previous studies in primary cultured rat hepatocytes showing that a much higher concentration of choline than ethanolamine is required for maximal stimulation of PC synthesis, which might be due to the rapid irreversible oxidation of choline to betaine (34).

It has been shown that Oct1-deficient mice are characterized by a normal systemic level of thiamine and hepatic thiamine deficiency. It has been proposed that under hepatic thiamine deficiency, hepatocytes switch from glycolysis to fatty acid oxidation, which in turn protects from hepatic steatosis (23). Thiamine plasma concentration in mice is approximately 0.1 μmol/L (36). Thiamine serum level in Oct1/2^−/−^ mice is 45 times higher than that in the WT mice (Fig. 1*A*), a concentration at which the thiamine transporter-1 (SLC19A2) still operates far from saturation (K_t_ = 2.5 μmol/L) (37, 38) and arguably compensate for the lack of Oct1. Indeed, although the thiamine hepatic content could not be assessed for this cohort, it has been previously shown that despite the stark elevation in circulating thiamine, Oct1/2^−/−^ mice hepatic content of thiamine is similar to that measured in the WT mice, hence do not experience hepatic thiamine deficiency (39). The different level of thiamine between Oct1^−/−^ and Oct1/2^−/−^ mice and the respective WT control mice stems from the well-known pronounced mutual redundancy between Oct1 and Oct2 in cation renal tubular secretion (24) and underscores the importance of active tubular secretion in thiamine systemic homeostasis (40).

The present study represents the first molecular characterization of ethanolamine membrane transport and demonstrates that systemic ethanolamine homeostasis depends on OCT1/2 function. Considering the established and emerging roles of PE in lipid droplet formation (41), very-low-density lipoprotein metabolism (42, 43, 44), and atherosclerosis (42), our findings demand rigorous future investigations to evaluate the response of mice lacking Oct1 and Oct2 with respect to hepatic function, plasma lipoprotein profile, and development of atherosclerosis, especially when fed a high-fat diet.

## Experimental procedures

### Reagents

Dulbecco’s modified Eagle medium, fetal bovine serum, Penicillin, Streptomycin and Geneticin G418 were acquired from Thermo Fisher Scientific. Ethanolamine [1-^3^H] HCl ([^3^H]ethanolamine, specific activity: 60 Ci/mmol) was purchased from ANAWA Trading AG, nonlabeled ethanolamine from Sigma-Aldrich. Poly-D-Lysine was acquired from Corning.

### Animals

Animals were kept under temperature-, light-, and humidity-controlled conditions with free access to water and standard mice chow (Altromin, Lage). Metabolomics and lipidomics were performed from leftover serum from previous experiments performed with untreated male mice. Experiments were approved by a governmental committee on animal welfare and were performed in accordance with national animal protection laws. Twelve-week-old (9–14 weeks) WT FVB/NJ mice (Harlan-Winkelmann) and 23-week-old (18–30 weeks) Oct1/2^−/−^ FVB/NJ mice (Prof. Schinkel, The Netherlands Cancer Institute) were used. Control experiments were performed with 10-week-old WT (Animal Facility of the Medical Faculty in Muenster) and 25-week-old (16–28 weeks) Oct2^−/−^ (Prof. Schinkel, The Netherlands Cancer Institute) C57BL/6 male mice. The Oct1/2^−/−^ and the Oct2^−/−^ mice have been described in detail elsewhere (24). The colonies of the Oct1/2^−/−^ and the Oct2^−/−^ mice were refreshed every 10 generations to maintain genetic integrity.

### Cell lines

WT (CRL-1573, American Type Culture Collection) and stably transfected HEK cortex 293 cells (HEK293) were cultured in Dulbecco’s Modified Eagle Medium containing 1 g/L D-glucose and supplemented with 10% (v/v) fetal bovine serum, 100 U/ml penicillin, 100 μg/ml streptomycin, at 37 °C in a humidified atmosphere of 5% CO_2_. HEK293 cells stably expressing the pcDNA3.1 plasmid containing the untagged coding sequence of OCT1, OCT2, Oct1, or Oct2 were grown under antibiotic selection with Geneticin G418 at the extracellular concentration of 400 μg/ml (12, 45, 46, 47, 48). All cell lines were consistently tested and confirmed negative for *mycoplasma* contamination throughout the duration of the project.

### Transport assay

Cells were seeded in 35-mm dishes precoated with 0.1 mg/ml Poly-D-Lysine at a density of 5 × 10^5^ cells per well/dish and transport experiment performed 48 to 72 h after seeding. Uptake of ethanolamine was measured using a standard protocol for rapid uptake determination in adherent cells (47, 49). Cells were washed and equilibrated with prewarmed (37 °C) transport buffer (5.3 mM KCl, 1 mM NaH_2_PO_4_, 0.8 mM MgSO_4_, 5.5 mM D-Glucose, 20 mM Hepes, 116.4 mM NaCl, adjusted to pH 7.4 with NaOH). The uptake was initiated by adding 0.5 ml of prewarmed (37 °C) transport buffer containing 0.2 to 0.6 μCi/ml [^3^H]ethanolamine and nonlabeled ethanolamine to achieve the desired final concentration. Uptake was terminated by rapid aspiration of the incubation solution followed by 8 to 10 washing steps with ice-cold transport buffer. Cells were lysed in 1 ml of 1% (w/v) Triton X-100 in dH_2_O. Five hundred microliters of the lysate were used for liquid scintillation counting. Twenty-five microliters of lysate were used for protein determination by bicinchoninic acid protein assay (Interchim, Montluçon Cedex). The counts per minute were normalized for the specific activity of the transport solution to calculate the intracellular level of ethanolamine. The OCT-specific transport was obtained by subtracting the intracellular level of ethanolamine in WT HEK293 cells.

### Serum targeted metabolomics analysis

To explore potential metabolic consequences of Oct1/2 deficiency, serum samples from Oct1/2^−/−^ and WT mice were analyzed using a targeted LC-MS/MS workflow. This approach combined the analysis of a broad set of metabolites, including amino acid derivatives, organic acids, acetyl-carnitines, and sugar derivatives, based on predefined multiple reaction monitoring (MRM) transitions from in-house spectral libraries. Serum sample preparation, extraction, and chromatographic conditions followed the procedure recommended for the Chromsystems amino acid analysis—Plasma/Serum kit (Chromsystems), ensuring methodological consistency. The combined targeted workflow allowed large metabolite screening and the absolute quantification of 48 amino acids included in the kit, which was further integrated with five additional derivatives: urea, creatinine, serotonin, and symmetric and asymmetric dimethylarginine. Briefly, 10 μl of serum was mixed with labeled internal standards, followed by protein precipitation using the Chromsystems precipitation reagent. After centrifugation, the clear supernatant was transferred to autosampler vials for LC-MS/MS analysis. Chromatographic separation was performed on the hydrophilic interaction liquid chromatography column supplied with the kit under gradient conditions, with an injection volume of 2 μl and a total run time of approximately 20 min. Quantification was achieved using MRM on a triple quadrupole LC-MS/MS system (AB Sciex 6500 QTRAP) equipped with a turbo electrospray ionization (ESI) source in positive ion mode. The specific MRM transitions used for the additional metabolites are reported as Supporting Information. Calibration curves for each amino acid and derivative were generated using three calibrators supplied by the manufacturer, and quality control samples at low, medium, and high concentrations were analyzed in parallel to assess accuracy and system performance. Data acquisition and processing were performed using MultiQuant software (version 3.3) (https://sciex.com/products/software/multiquant-software), with peak integration and quantification based on internal standard normalization. In total, 48 amino acids and five additional derivatives were quantified, and concentrations are expressed in μmol/L.

### Targeted serum lipidomics profiling

A targeted lipidomic analysis was performed using LC-MS/MS on an Ultivo triple quadrupole mass spectrometer (Agilent Technologies), following the method described by Weir *et al.* (2013) with minor modifications (50). Briefly, lipids from 20 μl of serum were extracted using 300 μl of chloroform/methanol (1:2, v/v). Samples were vortexed for 30 s, sonicated for 20 min, and then centrifuged at 23,000*g* for 10 min at 4 °C. A 5-μl aliquot of the resulting supernatant was directly injected into the LC-MS/MS system. Chromatographic separation was achieved using a Zorbax Eclipse Plus C18 column (2.1 × 50 mm, 1.8 μm; Agilent Technologies), maintained at 60 °C. The flow rate was set to 0.2 ml/min, and a binary solvent system was used: mobile phase A consisted of 5 mM ammonium formate in dH_2_O, and mobile phase B consisted of 50% isopropanol/50% methanol containing 5 mM ammonium formate. The initial elution gradient at 80% A/20% B was held for 3 min and then linearly ramped to reach a 100% B at 10 min. At 10.1 min, the flow rate increased to 0.5 ml/min, and 100% B was maintained until 12 min. Re-equilibration to initial conditions (80% A/20% B) was completed by minute 17. Lipid species eluting from the column were analyzed with the mass spectrometer operating in both positive and negative ESI modes. The ESI source parameters were capillary voltage +6000 V/−3500 V, nozzle voltage +1000 V/0 V, drying gas temperature 200 °C, gas flow 10 L/min, nebulizer pressure 35 psi, sheath gas temperature 350 °C, and sheath gas flow 11 L/min. MRM was used for targeted detection and quantification of 438 lipid species across multiple classes, including: PCs (n = 90), PEs (n = 39), sphingomyelins (n = 36), ceramides (n = 18), lysophospholipids such as lysophosphatidylcholines (n = 27) and LPEs (n = 6), DGs (n = 59), triacylglycerols (n = 43), free fatty acids (n = 41), cholesterol esters (n = 26), cholesterol, hexosylceramides (n = 24), phosphatidylglycerols (n = 4), phosphatidylinositols (n = 17), and phosphatidylserines (n = 7). MRM signal intensities were first normalized to the total ion current for each sample. Subsequently, each lipid variable was log-transformed and z-score normalized prior to statistical analysis.

### Data analysis

Metabolomics and lipidomics data were analyzed with MetaboAnalyst 6.0 and plotted using the open-access language programming software R Version 4.5.0 (R Project for Statistical Computing) or GraphPad Prism version 10 (https://www.graphpad.com). Transport data were analyzed and plotted with GraphPad Prism version 10. Details on the statistical analysis are provided in each figure legend.

## Data availability

All data, including the metabolomics and lipidomics datasets, are contained within the article and the supplementary information available online.

## Supporting information

This article contains supporting information.

## Conflict of interest

The authors declare that they have no conflicts of interest with the contents of this article.

## References

[1] Koepsell H. (2013). The SLC22 family with transporters of organic cations, anions and zwitterions. Mol. Aspects Med..

[2] Yee S.W., Buitrago D., Stecula A., Ngo H.X., Chien H.C., Zou L. (2020). Deorphaning a solute carrier 22 family member, SLC22A15, through functional genomic studies. FASEB J..

[3] Galluccio M., Mazza T., Scalise M., Sarubbi M.C., Indiveri C. (2022). Bacterial over-expression of functionally active human CT2 (SLC22A16) carnitine transporter. Mol. Biol. Rep..

[4] Khanppnavar B., Maier J., Herborg F., Gradisch R., Lazzarin E., Luethi D. (2022). Structural basis of organic cation transporter-3 inhibition. Nat. Commun..

[5] Zeng Y.C., Sobti M., Quinn A., Smith N.J., Brown S.H.J., Vandenberg J.I. (2023). Structural basis of promiscuous substrate transport by Organic Cation Transporter 1. Nat. Commun..

[6] Koepsell H. (2019). Multiple binding sites in organic cation transporters require sophisticated procedures to identify interactions of novel drugs. Biol. Chem..

[7] Xiu F., Rausch M., Gai Z., Su S., Wang S., Visentin M. (2023). The role of organic cation transporters in the pharmacokinetics, pharmacodynamics and drug-drug interactions of tyrosine kinase inhibitors. Int. J. Mol. Sci..

[8] Samodelov S.L., Kullak-Ublick G.A., Gai Z., Visentin M. (2020). Organic cation transporters in human physiology. Pharmacol. Toxicol. Int. J. Mol. Sci..

[9] Nies A.T., Koepsell H., Winter S., Burk O., Klein K., Kerb R. (2009). Expression of organic cation transporters OCT1 (SLC22A1) and OCT3 (SLC22A3) is affected by genetic factors and cholestasis in human liver. Hepatology.

[10] Zhang L., Dresser M.J., Gray A.T., Yost S.C., Terashita S., Giacomini K.M. (1997). Cloning and functional expression of a human liver organic cation transporter. Mol. Pharmacol..

[11] Motohashi H., Sakurai Y., Saito H., Masuda S., Urakami Y., Goto M. (2002). Gene expression levels and immunolocalization of organic ion transporters in the human kidney. J. Am. Soc. Nephrol. : JASN.

[12] Visentin M., Torozi A., Gai Z., Hausler S., Li C., Hiller C. (2018). Fluorocholine transport mediated by the organic cation transporter 2 (OCT2, SLC22A2): implication for imaging of kidney tumors. Drug. Metab. Dispos..

[13] Tzvetkov M.V., Matthaei J., Pojar S., Faltraco F., Vogler S., Prukop T. (2018). Increased systemic exposure and stronger cardiovascular and metabolic adverse reactions to fenoterol in individuals with heritable OCT1 deficiency. Clin. Pharmacol. Ther..

[14] Fukuda T., Chidambaran V., Mizuno T., Venkatasubramanian R., Ngamprasertwong P., Olbrecht V. (2013). OCT1 genetic variants influence the pharmacokinetics of morphine in children. Pharmacogenomics.

[15] Tzvetkov M.V., Saadatmand A.R., Bokelmann K., Meineke I., Kaiser R., Brockmoller J. (2012). Effects of OCT1 polymorphisms on the cellular uptake, plasma concentrations and efficacy of the 5-HT(3) antagonists tropisetron and ondansetron. Pharmacogenomics J..

[16] Tzvetkov M.V., Saadatmand A.R., Lotsch J., Tegeder I., Stingl J.C., Brockmoller J. (2011). Genetically polymorphic OCT1: another piece in the puzzle of the variable pharmacokinetics and pharmacodynamics of the opioidergic drug tramadol. Clin. Pharmacol. Ther..

[17] Shu Y., Sheardown S.A., Brown C., Owen R.P., Zhang S., Castro R.A. (2007). Effect of genetic variation in the organic cation transporter 1 (OCT1) on metformin action. J. Clin. Invest..

[18] Filipski K.K., Mathijssen R.H., Mikkelsen T.S., Schinkel A.H., Sparreboom A. (2009). Contribution of organic cation transporter 2 (OCT2) to cisplatin-induced nephrotoxicity. Clin. Pharmacol. Ther..

[19] Neuhoff S., Ungell A.L., Zamora I., Artursson P. (2003). pH-dependent bidirectional transport of weakly basic drugs across Caco-2 monolayers: implications for drug-drug interactions. Pharm. Res..

[20] Koepsell H. (2020). Organic cation transporters in health and disease. Pharmacol. Rev..

[21] Pelis R.M., Wright S.H. (2014). SLC22, SLC44, and SLC47 transporters--organic anion and cation transporters: molecular and cellular properties. Cur. Top. Membr..

[22] Liang X., Yee S.W., Chien H.C., Chen E.C., Luo Q., Zou L. (2018). Organic cation transporter 1 (OCT1) modulates multiple cardiometabolic traits through effects on hepatic thiamine content. Plos Biol..

[23] Chen L., Shu Y., Liang X., Chen E.C., Yee S.W., Zur A.A. (2014). OCT1 is a high-capacity thiamine transporter that regulates hepatic steatosis and is a target of metformin. Proc. Natl. Acad. Sci. U. S. A.

[24] Jonker J.W., Wagenaar E., Van Eijl S., Schinkel A.H. (2003). Deficiency in the organic cation transporters 1 and 2 (Oct1/Oct2 [Slc22a1/Slc22a2]) in mice abolishes renal secretion of organic cations. Mol. Cell. Biol..

[25] Ciarimboli G., Lancaster C.S., Schlatter E., Franke R.M., Sprowl J.A., Pavenstadt H. (2012). Proximal tubular secretion of creatinine by organic cation transporter OCT2 in cancer patients. Clin. Cancer Res..

[26] van der Veen J.N., Kennelly J.P., Wan S., Vance J.E., Vance D.E., Jacobs R.L. (2017). The critical role of phosphatidylcholine and phosphatidylethanolamine metabolism in health and disease. Biochim. Biophys. Acta Biomembr..

[27] Yorek M.A., Dunlap J.A., Spector A.A., Ginsberg B.H. (1986). Effect of ethanolamine on choline uptake and incorporation into phosphatidylcholine in human Y79 retinoblastoma cells. J. Lipid Res..

[28] Pu G.A., Anderson R.E. (1984). Ethanolamine accumulation by photoreceptor cells of the rabbit retina. J. Neurochem..

[29] Zelinski T.A., Choy P.C. (1984). Ethanolamine inhibits choline uptake in the isolated hamster heart. Biochim. Biophys. Acta.

[30] Lipton B.A., Yorek M.A., Ginsberg B.H. (1988). Ethanolamine and choline transport in cultured bovine aortic endothelial cells. J. Cell. Physiol..

[31] Grassl S.M. (2001). Ethanolamine transport in human placental brush-border membrane vesicles. J. Pharmacol. Exp. Ther..

[32] Houweling M., Tijburg L.B., Vaartjes W.J., van Golde L.M. (1992). Phosphatidylethanolamine metabolism in rat liver after partial hepatectomy. Control of biosynthesis of phosphatidylethanolamine by the availability of ethanolamine. Biochem. J..

[33] Gibellini F., Smith T.K. (2010). The Kennedy pathway--De novo synthesis of phosphatidylethanolamine and phosphatidylcholine. IUBMB Life.

[34] Sundler R., Akesson B. (1975). Regulation of phospholipid biosynthesis in isolated rat hepatocytes. Effect of different substrates. J. Biol. Chem..

[35] Tijburg L.B., Houweling M., Geelen J.H., van Golde L.M. (1987). Stimulation of phosphatidylethanolamine synthesis in isolated rat hepatocytes by phorbol 12-myristate 13-acetate. Biochim. Biophys. Acta.

[36] Xu H., Liu D., Chen J., Li H., Xu M., Wen W. (2019). Effects of chronic voluntary alcohol drinking on thiamine concentrations, endoplasmic reticulum stress, and oxidative stress in the brain of crossed high alcohol preferring mice. Neurotox Res..

[37] Oishi K., Hirai T., Gelb B.D., Diaz G.A. (2001). Slc19a2: cloning and characterization of the murine thiamin transporter cDNA and genomic sequence, the orthologue of the human. TRMA Gene Mol. Genet. Metab..

[38] Dutta B., Huang W., Molero M., Kekuda R., Leibach F.H., Devoe L.D. (1999). Cloning of the human thiamine transporter, a member of the folate transporter family. J. Biol. Chem..

[39] Kato K., Moriyama C., Ito N., Zhang X., Hachiuma K., Hagima N. (2015). Involvement of organic cation transporters in the clearance and milk secretion of thiamine in mice. Pharm. Res..

[40] Rennick B.R. (1958). The renal tubular excretion of choline and thiamine in the chicken. J. Pharmacol. Exp. Ther..

[41] Pol A., Gross S.P., Parton R.G. (2014). Review: biogenesis of the multifunctional lipid droplet: lipids, proteins, and sites. J. Cel. Biol..

[42] Zhao Y., Su B., Jacobs R.L., Kennedy B., Francis G.A., Waddington E. (2009). Lack of phosphatidylethanolamine N-methyltransferase alters plasma VLDL phospholipids and attenuates atherosclerosis in mice. Arterioscler. Thromb. Vasc. Biol..

[43] Agren J.J., Kurvinen J.P., Kuksis A. (2005). Isolation of very low density lipoprotein phospholipids enriched in ethanolamine phospholipids from rats injected with Triton WR 1339. Biochim. Biophys. Acta.

[44] Leonardi R., Frank M.W., Jackson P.D., Rock C.O., Jackowski S. (2009). Elimination of the CDP-ethanolamine pathway disrupts hepatic lipid homeostasis. J. Biol. Chem..

[45] Thevenod F., Ciarimboli G., Leistner M., Wolff N.A., Lee W.K., Schatz I. (2013). Substrate- and cell contact-dependent inhibitor affinity of human organic cation transporter 2: studies with two classical organic cation substrates and the novel substrate cd2+. Mol. Phar..

[46] Schlatter E., Klassen P., Massmann V., Holle S.K., Guckel D., Edemir B. (2014). Mouse organic cation transporter 1 determines properties and regulation of basolateral organic cation transport in renal proximal tubules Pflugers Archiv. Eur. J. Physiol..

[47] Visentin M., van Rosmalen B.V., Hiller C., Bieze M., Hofstetter L., Verheij J. (2017). Impact of Organic Cation Transporters (OCT-SLC22A) on differential diagnosis of intrahepatic lesions. Drug Metab. Dispos..

[48] Mulgaonkar A., Venitz J., Grundemann D., Sweet D.H. (2013). Human organic cation transporters 1 (SLC22A1), 2 (SLC22A2), and 3 (SLC22A3) as disposition pathways for fluoroquinolone antimicrobials. Antimicrob. Agents Chemother..

[49] Hormann S., Gai Z., Kullak-Ublick G.A., Visentin M. (2020). Plasma membrane cholesterol regulates the allosteric binding of 1-Methyl-4-Phenylpyridinium to organic cation transporter 2 (SLC22A2). J. Pharmacol. Exp. Ther..

[50] Weir J.M., Wong G., Barlow C.K., Greeve M.A., Kowalczyk A., Almasy L. (2013). Plasma lipid profiling in a large population-based cohort. J. Lipid Res..

