# Supplementary material for: The organic cation transporters 1 and 2 mediate ethanolamine cellular efflux and control systemic phosphatidylethanolamine level
Source: J Biol Chem. 2025 Nov 5;301(12):110911. doi: 10.1016/j.jbc.2025.110911 (PMC12719660; doi:10.1016/j.jbc.2025.110911)
Supplement: Supporting Figures [file mmc2.docx]

**The organic cation transporters 1 and 2 mediate ethanolamine cellular efflux and control systemic phosphatidylethanolamine level**

Julia Schubert, Ferhat Koca, Francesca Barone, Giuseppe Corona, Giuliano Ciarimboli, Michele Visentin

**Material Included**

**MRM transitions used for the additional metabolites**

**Supplementary Figure 1**

**Supplementary Figure 2**

**MRM transitions used for the additional metabolites**

Urea: 61.0 → 44.1 m/z (IS: 15N2-Urea 63.0 → 45.1 m/z)

Creatinine: 114 → 86.1 m/z (IS: 3d-Creatinine 117.0 → 47.0 m/z)

Serotonin: 177.1 → 160.1 m/z (IS: 4d-Serotonin 181.1 → 164.1 m/z)

SDMA: 203.2 → 172.2 m/z (IS: 6d-SDMA 209.2 → 175.2 m/z)

ADMA: 203.2 → 46.2 m/z (IS: 6d-ADMA 209.1 → 52.2 m/z)

**Figure S1. Ethanolamine level in Oct2^−/−^ mice.** Scatter dot plots of the absolute abundance of ethanolamine in the serum of wild type and Oct2^-/-^ 57BL/6e mice. Each dot represents one individual sample. Comparisons of the means were performed by unpaired Student's t-test.


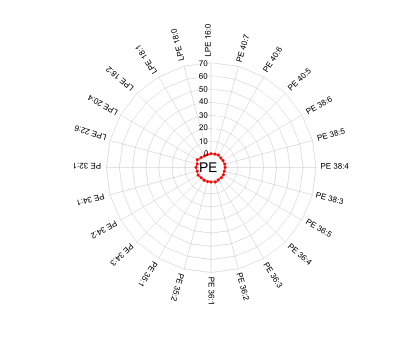


**Figure S2. Radar plot of ethanolamine-derived lipid species in Oct2^−/−^ mice**. Each axis represents a lipid species and the length of each axis corresponds to the fold change between the values measured in the Oct2^-/-^ relative to those measured in the WT mice. The plot was scale-matched to the Oct1/2^-/-^/WT ratios shown in figure 6.
